# Diversity of Clinical and Molecular Characteristics in Korean Patients with 16p11.2 Microdeletion Syndrome

**DOI:** 10.3390/ijms25010253

**Published:** 2023-12-23

**Authors:** Ji Yoon Han, Yong Gon Cho, Dae Sun Jo, Joonhong Park

**Affiliations:** 1 Department of Pediatrics, College of Medicine, The Catholic University of Korea, Seoul 06591, Republic of Korea; han024@catholic.ac.kr; 2 Department of Laboratory Medicine, Jeonbuk National University Medical School and Hospital, Jeonju 54907, Republic of Korea; choyg@jbnu.ac.kr; 3 Research Institute of Clinical Medicine of Jeonbuk National University-Biomedical Research Institute of Jeonbuk National University Hospital, Jeonju 54907, Republic of Korea; 4 Department of Pediatrics, Jeonbuk National University Medical School and Hospital, Jeonju 54907, Republic of Korea

**Keywords:** 16p11.2 microdeletion, phenotypic heterogeneity, CytoScan Dx Assay, neonatal hypotonia, benign infantile epilepsy, developmental delay, intellectual disability, vertebral abnormalities

## Abstract

16p11.2 copy number variations (CNVs) are increasingly recognized as one of the most frequent genomic disorders, and the 16p11.2 microdeletion exhibits broad phenotypic variability and a diverse clinical phenotype. We describe the neurodevelopmental course and discordant clinical phenotypes observed within and between individuals with identical 16p11.2 microdeletions. An analysis with the CytoScan Dx Assay was conducted on a GeneChip System 3000Dx, and the sample signals were then compared to a reference set using the Chromosome Analysis Suite software version 3.1. Ten patients from six separate families were identified with 16p11.2 microdeletions. Nine breakpoints (BPs) 4-5 and one BP2-5 of the 16p11.2 microdeletion were identified. All patients with 16p11.2 microdeletions exhibited developmental delay and/or intellectual disability. Sixty percent of patients presented with neonatal hypotonia, but muscle weakness improved with age. Benign infantile epilepsy manifested between the ages of 7–10 months (a median of 8 months) in six patients (60%). Vertebral dysplasia was observed in two patients (20%), and mild scoliosis was noted in three patients. Sixty percent of patients were overweight. We present six unrelated Korean families, among which identical 16p11.2 microdeletions resulted in diverse developmental trajectories and discordant phenotypes. The clinical variability and incomplete penetrance observed in individuals with 16p11.2 microdeletions remain unclear, posing challenges to accurate clinical interpretation and diagnosis.

## 1. Introduction

Highly homologous blocks of low copy repeats (LCRs) in the pericentromeric region of 16p11 can serve as substrates for non-allelic homologous recombination, potentially predisposing individuals to genomic disorders that contribute to instability and a susceptibility to recurrent copy number variations (CNVs) [1]. Within the 16p11.2 locus, five LCRs have been identified as mediators of recurrent and clinically significant imbalances within the 16p11.2 chromosomal band. These ‘recombination hotspots’ from telomere to centromere were proposed as breakpoints BP1 to BP5. Typical 16p11.2 CNVs arise from recurring proximal deletions or duplications spanning approximately 600 kb, which are delineated by BP4 and BP5 at genome sequence coordinates 29.5 and 30.1 Mb, or distal rearrangements mediated by BP1-BP3 and BP2-BP3, spanning 550 kb and 220 kb, respectively, and containing the *SH2B1* gene, which have also been documented in individuals with early-onset obesity and variable degrees of developmental delay (DD) [2]. Several recurrent rearrangements overlap with the proximal BP4-BP5 region, including the 1.7 Mb deletions and duplications from BP1 to BP5, which should be considered distinct entities. [3]. Due to the extensive use of chromosomal microarrays (CMAs) in clinical settings, 16p11.2 CNVs are now being more commonly identified and are considered one of the more prevalent genomic disorders [4,5]. In the general population, the occurrence of 16p11.2 microdeletions have been noted at a frequency ranging from approximately 0.028% to 0.043%, while duplications are observed in the range of 0.035% to 0.053% [6,7]. In particular, 16p11.2 microdeletions affect nearly 1 in 200 patients with autistic spectrum disorder (ASD) and approximately 1 in 1000 patients with language or neuropsychiatric disorders [8,9]. 16p11.2 microdeletion syndrome has also been associated with DD, intellectual disability (ID), and neuropsychiatric disorders (including ASD) in children [10]. The 16p11.2 microdeletion exhibits broad phenotypic variability and a diverse clinical phenotype. Seizures occur in approximately 25% of patients, while neonatal hypotonia, obesity, vertebral abnormalities, sensorineural hearing loss, macrocephaly, and cardiovascular abnormalities have been reported in some patients [11,12].

Several genetic studies of patients with 16p11.2 microdeletion syndrome, identifying phenotypic heterogeneity in different ethnic populations, have been documented [13,14,15]. In this study, we describe the neurodevelopmental course and discordant clinical phenotypes observed within and between Korean individuals with identical 16p11.2 microdeletions. We identify two kinds of 16p11.2 microdeletions using the Affymetrix CytoScan Dx Assay (Santa Clara, CA, USA). To the best of our knowledge, this is the first study of Korean patients with 16p11.2 microdeletions comparing phenotypic differences across unrelated families.

## 2. Results

**Figure 1:**
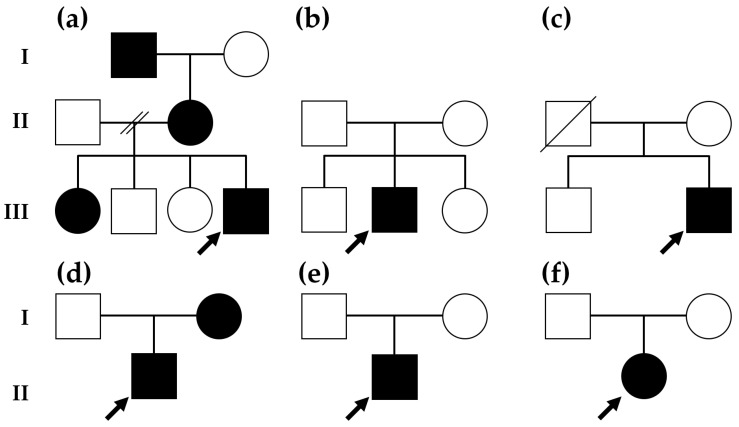
Pedigree analyses were conducted on ten Korean patients with 16p11.2 microdeletion syndrome. Among the six probands (indicated by black arrows), two probands (**a**,**d**) inherited the condition from their mothers, while the remaining four patients (**b**,**c**,**e**,**f**) were affected by a new mutation.

Patients were referred by physicians as part of a clinical assessment for DD/ID, hypotonia, epilepsy, ASD, or a co-occurrence of these clinical manifestations with unexplained etiology. The Autism Spectrum Screening Questionnaire (ASSQ) and Childhood Autism Rating Scale, 2nd Edition (CARS2) were employed as screening tools for identifying ASD and DD in infants and young children. ASD was diagnosed according to the Diagnostic and Statistical Manual of Mental Disorders, Fifth Edition (DSM-5). G-banded karyotyping and CMA were performed sequentially, but if G-banded karyotyping was normal, CMA was conducted as the first-tier diagnostic cytogenetic testing. The advancement of CMA has facilitated the elucidation of apparently atypical phenotypes in certain aneuploidies by identifying additional CNVs that conventional karyotyping fails to detect [16]. The origin of any CNV identified in the proband was determined through a paternity analysis of parental samples. As a result, ten patients from six separate families were diagnosed as having 16p11.2 microdeletion syndrome. Four patients experienced de novo occurrences, while another four patients had inherited symptoms from symptomatic parents. It is not known whether the parents (A-I-1 and D-I-2 in Figure 1a and 1d, respectively) of the patient with a family history had inherited the genetic condition or not. The second gross rearrangement was identified in two patients: B-II-2 with 11q14.1 duplication and D-I-2 with 17q21.31 deletion. Pure trisomy 11q cases are defined as cases with minimal or no involvement of another chromosome. Among them, four trisomy 11q cases involved almost the entire long arm of chromosome 11, with a common region of 11q14-q24. All applicable patients exhibited dysmorphic facial features, short stature/growth retardation, DD, seizures, congenital heart defects, and urogenital and extremity abnormalities. The large number of genes and dosage effects of these genes are associated with the relatively severe phenotypes observed in these cases. On the other hand, there were two cases of trisomy 11q resulting from interstitial duplications in the proximal region of 11q13-q14. Both patients exhibited nonspecific craniofacial features and skeletal anomalies, as well as mild-to-moderate DD [17]. Details of 16p11.2 microdeletions and other CNVs in ten Korean patients with 16p11.2 microdeletion syndrome are summarized in Table 1.

**Figure 2:**
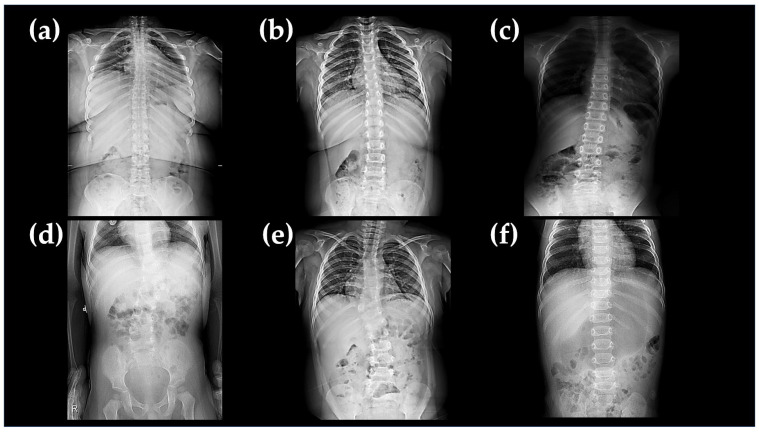
Radiologic findings indicate vertebral abnormalities, including scoliosis and vertebral dysplasia, in Korean patients with 16p11.2 microdeletion syndrome. Three patients, A-III-1 (**a**), A-III-4 (**b**), and B-II-2 (**c**), exhibit scoliosis, while two patients, D-II-1 (**d**) and E-II-1 (**e**), present with vertebral dysplasia. (**f**) Patient F-II-1 had a normal lumbar spine X-ray.

All patients with the 16p11.2 microdeletion exhibited DD and/or ID. Six out of ten patients presented with neonatal hypotonia, but muscle weakness improved with age, and benign infantile epilepsy (BIE) manifested between the ages of 7–10 months (a median of 8 months). Among them, two patients (A-I-1 and C-II-2 in Figure 1a and 1c, respectively) were diagnosed as epilepsy during childhood. Most seizures were effectively controlled with oxcarbazepine and/or levetiracetam. Vertebral dysplasia was observed in two patients, and mild scoliosis was noted in three patients (Figure 2). Six out of ten patients were overweight, and one patient was given an ASD diagnosis at 6 years old (B-2-II in Figure 1b). Renal anomalies are uncommon features in patients with 16p11.2 microdeletion, despite the known association of kidney malformations with the 16p11.2 distal deletion [18]. In patients with obesity, we conducted abdominal sonograms, including assessments of the liver, spleen, and kidneys, to evaluate fatty liver. The sonogram results were normal in all cases. Brain magnetic resonance imaging (MRI) studies were conducted on seven patients, revealing normal results, including structures and myelination. The interictal electroencephalography (EEG) results were mostly normal without epileptiform discharges, with the exception of one patient (C-II-2 in Figure 1c). The mean intelligence quotient (IQ) of the studied patients was 65.5, ranging from 60 to 69, corresponding to mild ID. The clinical phenotypes of ten Korean patients with 16p11.2 microdeletion syndrome are summarized in Table 2.

### 2.1. Patient Description

#### 2.1.1. Family A

A 6-year-old boy (A-III-4 in Figure 1a) had poor motor control until the age of 3 months, after which there was significant improvement around 15 months. He achieved independent walking at 18 months. At approximately 2 years of age, he experienced three episodes of simple febrile seizures. His IQ was estimated at 62, indicating mild ID at 5 years old. He did not exhibit dysmorphic features or behavioral disabilities. A brain MRI revealed a normal myelination pattern without obvious abnormalities. At 6 years old, his body weight was 29.4 kg, and his body mass index (BMI) was 25 kg/m^2^, placing him above the 97th percentile. Among the proband’s siblings, his first sister (A-III-1 in Figure 1a) was born at 38 weeks of gestational age, and the pregnancy was uneventful. Early signs of hypotonia appeared during the neonatal to early infancy period, but she showed improvement in motor function by 14 months, enabling her to walk. Seizures occurred at 8 months, and oxcarbazepine, as an anti-seizure medication (ASM), was administered as monotherapy in the treatment of epilepsy to manage focal seizures for a duration of 3 months. A brain MRI revealed normal structure, and interictal EEG findings displayed a normal pattern. She exhibited global DD/ID with an IQ of 65 at the age of seven. Severe obesity (BMI of 40 kg/m^2^) was observed at 13 years of age. Subsequent seizures did not occur after infancy, and mild scoliosis was noted on a lumbar spine X-ray. The proband’s mother (A-II-1 in Figure 1a) was born at 37 weeks of gestational age, and her pregnancy was uneventful. Although she graduated from high school, her academic performance was subpar. She obtained a low IQ of 65 and was overweight with a BMI of 35 kg/m^2^. Radiologic findings showed no skeletal abnormalities, and there were no suspicious facial dysmorphisms. The proband’s grandfather (A-I-1 in Figure 1a) experienced generalized tonic–clonic seizures in infancy and late childhood, requiring ongoing ASM (oxcarbazepine) until reaching adulthood. With an IQ of 69, signifying mild ID, and a BMI of 30 kg/m^2^, indicating obesity, he graduated from middle school with poor academic performance. Despite this, he secured employment as a clerk at a grocery store. Notably, there were no abnormalities in his appearance, and his musculoskeletal system exhibited no peculiarities.

#### 2.1.2. Patient B-II-2

B-II-2, a 5-year-old boy (Figure 1b), was delivered via cesarean section to non-consanguineous parents at 38 + 5 weeks, with a birth weight of 3.2 kg. Hypotonia was evident during early infancy, and there was gradual improvement in motor function by 18 months. Seizure activity became noticeable at 10 months of age but was self-limited without the need for ASM. Neurodevelopmental milestones were delayed; he began walking independently at 22 months and uttered his first words at 2.5 years. A profound language delay led to enrollment in a specialized kindergarten for speech therapy. Formal testing indicated ASD. He received a diagnosis of ID with an IQ of 67 at the age of 7. A brain MRI revealed normal structures, and skeletal imaging showed mild scoliosis. No suspicious facial dysmorphism was observed.

#### 2.1.3. Patient C-II-2

C-II-2, a 16-year-old boy (Figure 1c), was born at term to healthy parents. BIE, characterized by clustering seizures, occurred from infancy to 2 years old. Global DD, primarily affecting speech, was first noted at 3 years of age. His neuromotor development was slow, without autistic features. At the age of 12, he attended an elementary school with special educational programs. IQ testing yielded scores in the mild ID range (IQ of 68), and his BMI of 25 kg/m^2^ indicated that he was overweight at the age of 12. Seizures recurred at the age of 5 and were controlled with polytherapy (levetiracetam and oxcarbazepine). Intermittent fasting activities from both occipital areas were observed on an interictal EEG. He exhibited no autistic features or apparent facial dysmorphism. Learning problems and mild speech issues were noted. While he does not live independently, he has been able to perform simple tasks. His family history is negative for DD/ID or epilepsy.

#### 2.1.4. Family D

D-II-1, a 3-year-old boy (Figure 1d), was born at 37 weeks of gestational age with a birth weight of 2.65 kg. He lacked head control at the age of 4 months and began walking independently at 24 months. Vertebral dysplasia was diagnosed at 12 months. He exhibited delayed psychomotor development, and his vocabulary was limited, with an IQ of 60 at six years old; he is not able to read or write. His mother (D-I-1 in Figure 1d) was delivered normally. She exhibited mild ID with an IQ of 68 and was obese with a BMI of 30 kg/m^2^. Despite these conditions, she presented a healthy appearance without facial dysmorphism.

#### 2.1.5. Patient E-II-1

E-II-1, a 1-year-old boy (Figure 1e), was born via cesarean section at 38 + 5 weeks and was admitted to the neonatal intensive care unit due to seizures and hypotonia. A brain MRI revealed normal structures, and an interictal EEG showed a normal pattern appropriate for his age. A lumbar spine X-ray diagnosed congenital scoliosis, involving combined hemi-vertebrae at T11 and T12. During late infancy, there was a gradual improvement in motor function, and he achieved sitting without support at 9 months of age. Independent walking became possible at 16 months, with speech limited to several words at 20 months. At 8 months old, levetiracetam was administered to control clustered seizures. His family history is negative for both epilepsy and vertebral abnormalities.

#### 2.1.6. Patient F-II-1

F-II-1, a 1-year-old girl (Figure 1f), was born at 40 weeks of gestation by cesarean section, and the pregnancy was uneventful. Her development during early childhood was within the normal range. At the age of 6 months, she experienced multiple seizures and was admitted to the hospital. After the administration of levetiracetam and oxcarbazepine, the seizures ceased. A brain MRI reported normal myelination appropriate for her age, and no abnormal structures were observed. Her motor development was normal, starting to walk at 12 months of age, but language development was delayed. She did not exhibit dysmorphic features.

## 3. Discussion

The 16p11.2 microdeletion is strongly linked to ASD and ID, with DD and ASD being common presentations [19]. While the 16p11.2 microdeletion may manifest with poor height and weight development, as well as anorexia, it is more commonly associated with obesity and facial dysmorphism [20]. Furthermore, a series of case reports have indicated that cognitive and motor impairments are characteristic features, with ASD being one of the most prevalent phenotypes associated with this deletion. Speech impairment and epilepsy may also be observed in individuals with the 16p11.2 deletion [2]. This study specifically concentrated on a series of Korean cases referred for karyotyping that revealed interstitial deletions on chromosome 16p11.2. In our cases with CMA results, nine exhibited a deletion between BP4 and 5, while one had a deletion between BP2 and 5. In previous studies on 16p11.2 CNVs, deletions were commonly identified as de novo, while duplications were generally inherited [21]. However, in the current prenatal series, among the ten patients from six families with known inheritance, there was a notable occurrence of familial transmission. Specifically, 40% (4/10) of the deletion cases were determined to be inherited either maternally (*n* = 3) or paternally (*n* = 1), while another 40% occurred sporadically. The frequency of inherited deletions noted here is significantly higher than previously reported. Furthermore, testing of parents led to the identification of additional individuals affected by 16p11.2 CNVs. A majority (57%, 4/7) of the transmitting parents appeared to be healthy following full evaluations, underscoring the incomplete penetrance of 16p11.2 CNVs. The observed phenotypes were relatively mild when compared to the reported phenotypic characteristics of 16p11.2 microdeletions. This may be because individuals with 16p11.2 CNVs and severe phenotypes are less likely to reproduce, whereas those with less severe phenotypes may still have the ability to produce offspring.

**Figure 3:**
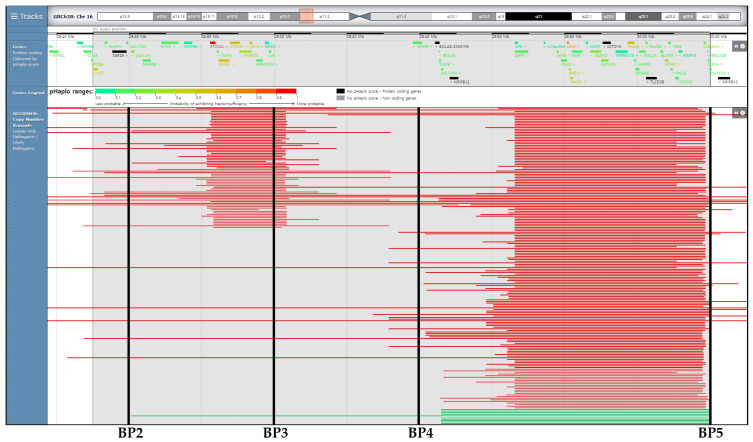
A schematic representation of Korean patients with 16p11.2 microdeletion syndrome is compared to previously reported cases spanning the interstitial 1.7 Mb of chromosome 16p11.2, as displayed by DECIPHER’s genome browser. Green bars indicate pathogenic losses of any size (in Mb) identified in our Korean patients with 16p11.2 microdeletion syndrome. Red bars on the browser indicate pathogenic losses of any size (in Mb), as classified by Decipher (https://www.deciphergenomics.org/browser#q/16:28,500,000-30,200,000/location/16:28375000-30325000; accessed on 11 October 2023). Abbreviation: BP, breakpoint.

While 16p11.2 rearrangements should not primarily be considered a malformation syndrome [10], we observed a seemingly elevated incidence of vertebral anomalies among proximal deletion fetuses, with 50% (5/10) displaying such anomalies. The common region of the 16p11.2 microdeletion occurs proximal to BP4-5 (chr 16:29,420,000–30,200,000) and is associated with the genetic interlude between *SULT1A1* and *SPN1*, encompassing 34 protein-coding genes (Figure 3). *TBX6* (T-box transcription factor 6) belongs to a group of highly conserved genes that share a common DNA-binding domain known as the T-box. T-box genes, including *TBX6*, encode transcription factors that play a crucial role in the regulation of developmental processes and have been suggested as susceptible genes for vertebral deformities [22]. Compound heterozygous mutations involving a null allele and a hypomorphic allele of *TBX6* have been identified as the cause of congenital scoliosis in one case series [23]. In this study, two patients (20%) exhibited vertebral anomalies and congenital scoliosis.

**Figure 4:**
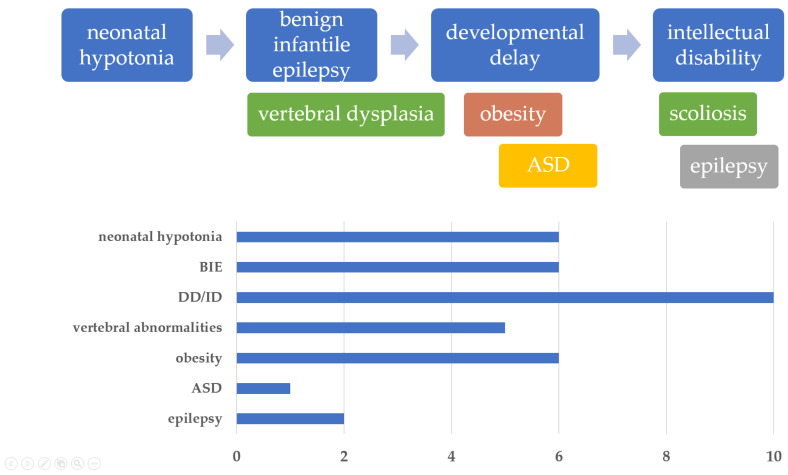
Overview of clinical phenotypes with age and illustration of symptom frequencies in Korean patients with 16p11.2 microdeletion syndrome. The sequence of main symptoms observed as the patient grows was indicated with arrows. The patient’s clinical manifestations were distinguished by color. Abbreviations: BIE, benign infantile epilepsy; DD/ID, developmental delay/intellectual disability; ASD, autism spectrum disorder.

The majority of individuals with the 16p11.2 microdeletion were identified during assessments for DD/ID. In most, if not all, cases, obesity (approximately 75%), ASD (20–25%), epilepsy/seizures (approximately 25%), vertebral anomalies (approximately 20%), or dysmorphic features were also noted [21]. However, systematic radiological and clinical assessments have not been conducted in most carriers. It is probable that only the most severe cases come to medical attention. Neurodevelopmental phenotypes associated with the 16p11.2 microdeletion, including ASD, are thought to be correlated with transcriptional profiles. The reported mean IQ for patients with the 16p11.2 microdeletion is 76 [4]; however, our results were 10 points lower at 65.5. Relevant signaling pathways may not be activated to the same extent in the brain and peripheral tissues [24,25,26]. The disruption or deletion of one allele of a gene may also produce a phenotypic effect through a recessive mechanism. This happens when, at the same locus as the deletion, a single-nucleotide variant or a CNV from the chromosome inherited from the other healthy parent is transmitted. This unmasking of a recessive allele constitutes a mixed mutation mechanism [27]. Deletion carriers from the same family provide an opportunity to understand the degree of phenotypic heterogeneity and to recognize potential genetic modifying factors, as they share significant elements of the genetic background while being exposed to similar environmental circumstances. Furthermore, genetic, epigenetic, and environmental factors can influence phenotypic diversity [28,29]. The presence of a CNV, especially one classified as pathogenic or likely pathogenic, might be considered an additional genetic factor contributing to the phenotypic heterogeneity among patients with 16p11.2 microdeletions, particularly in those exhibiting more severe ID. Therefore, patients with 16p11.2 microdeletions should be investigated for the coexistence of other genetic events. Some of our patients manifesting an atypical phenotype could be the recipients of a second pathogenic variant in the non-deleted allele or a second hit in the non-deleted allele, as has been reported for concurrent 16p11.2 microdeletions and other CNVs [30,31]. Examples of such include de novo CNVs in patients with an unusual clinical severity of 16p11.2 deletion syndrome, caused by an unmasked recessive mutation of CLN3 [32]. In this case, the patient’s clinical findings differed from the typical 16p11.2 microdeletion phenotypes and exhibited some features reminiscent of juvenile neuronal ceroid-lipofuscinosis. The 16p11.2 microdeletion predisposes individuals to obesity, with a 43-fold increased risk of morbid obesity [33]. In this study, the overall manifestation trajectory included, sequentially, neonatal hypotonia, BIE/vertebral dysplasia, ASD, DD/obesity, and ID/epilepsy/scoliosis. All participants exhibited DD and/or ID, with more than half presenting with neonatal hypotonia, epilepsy, and vertebral abnormalities. Only one patient was diagnosed with ASD, and none exhibited suspicious craniofacial abnormalities (Figure 4).

In a diploid genome, CNVs that affect the phenotype by altering the dosage of a gene exhibit a dominant effect. Haploinsufficiency or triplosufficiency for a gene due to the loss or gain of CNVs, respectively, may modify the stoichiometry of the proteins in a complex, thereby impacting its function [27]. A causal role of CNVs in the expression of quantitative trait loci uncovered a dosage compensation mechanism for certain transcript levels [34]. The pLI score is the probability that a given gene falls into the haploinsufficient category and, therefore, is extremely intolerant of loss-of-function (LoF) variation. Genes with high pLI scores (pLI ≥ 0.9) are extremely LoF intolerant, whereas genes with low pLI scores (pLI ≤ 0.1) are LoF tolerant [13]. Among protein-coding genes located between BP4 and 5, five genes, including *TAOK2* (a pLI score of 1.00), *CORO1A* (0.97), *MAZ* (0.93), *PAGR1* (0.74), and *PRRT2* (0.59), have a pLI value of 0.5 or more and are associated with symptoms related to 16p11.2 deficits and are implicated in genetic disease. Key roles for *MAPK3*, *KCTD13*, *MVP*, and *TAOK2* have been implicated in ASD. The *MAPK3* gene (encoding ERK1) and the major vault protein gene (*MVP*), both involved in the ERK/MAP kinase pathway, are associated with neurodevelopmental disorders [8,35]. Alterations within the *TAOK2*, *HIRIP3*, and *DOCA2* genes are considered potential causes of ASD, as they encode proteins critical for the appropriate development of neurons and, in particular, synaptic connectivity [36,37]. *KCTD13* is suggested to play a role in neuropsychiatric problems [38]. Golzio et al. [3] reported that *KCTD13* is a primary driver of neuronal proliferation and brain growth in zebrafish. In humans, the *KCTD13* gene is associated with development and an increase in BMI, linked with ID [39].

On the other hand, sequence variants of *PRRT2* leading to loss of function result in elevated intracellular glutamate levels and an increase in neuronal hyperexcitability [40]. Activity levels below 50% may contribute to disease, and different levels of PRRT2 activity could be associated with epilepsy [41]. Clinical phenotypes associated with *PRRT2* gene mutations primarily include BIE, paroxysmal kinesigenic dyskinesia, and choreoathetosis [42]. CNVs in the 16p11.2 region show an increased incidence of seizures and epilepsy compared to the 8% lifetime incidence of seizures in the general population [10,43]. CNVs at 16p11.2 involving the *PRRT2* gene have been reported as benign but have previously been linked to epilepsy, and mutations of *PRRT2* are associated with BIE [44,45]. In this study, 60% of patients experienced infantile seizures, and only two patients (20%) were diagnosed with epilepsy in childhood. The clinical heterogeneity observed could be attributed to a dominant negative effect of the *PRRT2* mutation or the contribution of modifier genes within the 16p11.2 microdeletion.

Genetic counseling for families at risk of having a child with 16p11.2 microdeletion presents challenges. The phenotypes associated with these variations are strongly linked to neuropsychiatric disorders, including DD/ID, ASD, and other psychiatric conditions, yet a normal outcome is also possible. For infantile cases, investigating mental and neurologic features is not feasible. Adding to the complexity, there appears to be variable expressivity among family members carrying CNVs [46]. In families A and D, we observed intra-familial discordance regarding the vertebral phenotype, where the affected mother showed no clinical findings consistent with vertebral defects. This underscores the genetic heterogeneity even within families. When interpreting the clinical significance of 16p11.2 microdeletions, it is crucial to provide prospective parents with comprehensive and detailed information regarding the incomplete penetrance and the range of potential phenotypic outcomes.

## 4. Materials and Methods

### 4.1. CytoScan Dx Assay

Genomic DNA (gDNA) was extracted from whole-blood samples obtained from patients who underwent karyotyping. The gDNA samples were processed at GC Genome Laboratories (Yongin, Republic of Korea) using the Affymetrix CytoScan Dx Assay. This CMA assay employs a high-density combined CGH and SNP array platform, evaluating approximately 2,696,550 markers, including around 750,000 SNPs and 1.9 million non-polymorphic markers. In brief, the analysis of the CytoScan Dx Assay was conducted on the GeneChip System 3000Dx (Thermo Fisher Scientific, Waltham, MA, USA), following the manufacturer’s instructions. The resulting array was scanned with a GeneChip Scanner, and the signal intensity of each marker was assessed. Using the Chromosome Analysis Suite (ChAS Dx) software version 3.1 (Thermo Fisher Scientific), the signals of the samples were then compared to a reference set based on the average of over 400 samples. Differences in the signals between the sample and reference were expressed as a log2 ratio, representing the relative intensity of each marker. In particular, the minimum size and number of markers for the reported CNVs are determined by the filter settings, which were set at 50 kb and 50 markers for CN gains and 25 kb and 25 markers for CN losses. A discrete copy number value was determined from the relative intensity data and displayed. Genotype information for the SNP markers was visualized with Allele Track.

### 4.2. Data Analysis

Using Human Genome Build 19 (Genome Reference Consortium GRCh37), the CytoScan Dx Assay CNV coordinates were compared with the coordinates of actionable microarray findings listed in the ClinGen (https://clinicalgenome.org/; accessed on 3 March 2023), Decipher (https://www.deciphergenomics.org/browser; accessed on 23 April 2023), Online Mendelian Inheritance in Man (OMIM, https://www.omim.org/; accessed on 9 April 2023), and dbVar (https://www.ncbi.nlm.nih.gov/dbvar/; accessed on 21 April 2023) databases. The CMA results were interpreted exclusively by healthcare professionals who are board-certified in molecular genetics or clinical cytogenetics. The CNVs were categorized into four groups: pathogenic, variants of possible significance (VOPS), variants of unknown significance (VOUS), and benign, according to American College of Medical Genetics standards and guidelines for interpretation and reporting of postnatal constitutional copy number variants [47]. Pathogenic or VOPS CNVs were considered abnormal. When available, known deletions/duplications identified via the CytoScan Dx Assay were confirmed by multiplex ligation-dependent probe amplification or fluorescence in situ hybridization.

## 5. Conclusions

Our study showed an overall common course of clinical manifestation with age; however, each patient demonstrated inter-individual and intra-familial variability. Due to the relative rarity of the disease, this report contributes to reinforcing a better phenotypic delineation of the 16p11.2 deletion. The clinical variability and incomplete penetrance observed in individuals with 16p11.2 microdeletions remain unclear, posing challenges to accurate clinical interpretation and diagnosis. Future studies involving larger cohorts of patients are essential to establishing a more profound understanding of the potential pathophysiology associated with the 16p11.2 microdeletion.

## Figures and Tables

**Table 1:** Details of 16p11.2 deletion and other copy number variations in Korean patients with 16p11.2 microdeletion syndrome.

Cases	SNP Microarray Results	Breakpoints	Inheritance
A-I-1	arr[hg19] 16p11.2(29,432,212–30,177,916) × 1	BP4–5	unknown
A-II-1	arr[hg19] 16p11.2(29,432,212–30,177,916) × 1	BP4–5	paternal
A-III-1	arr[hg19] 16p11.2(29,438,325–30,191,848) × 1	BP4–5	maternal
A-III-4	arr[hg19] 16p11.2(29,438,325–30,191,848) × 1	BP4–5	maternal
B-II-2	arr[hg19] 16p11.2(28,508,988–30,177,240) × 1	BP2–5	de novo
	arr[hg19] 11q14.1(80,587,297–81,818,908) × 3	na	de novo
C-II-2	arr[hg19] 16p.11.2(29,581,100–30,177,240) × 1	BP4–5	de novo
D-I-2	arr[hg19] 16p.11.2(29,567,295–30,177,916) × 1	BP4–5	unknown
	arr[hg19] 17q21.31(44,188,450–44,267,478) × 1	na	unknown
D-II-1	arr[hg19] 16p.11.2(29,609,934–30,177,240) × 1	BP4–5	maternal
E-II-1	arr[hg19] 16p.11.2(29,567,295–30,190,029) × 1	BP4–5	de novo
F-II-1	arr[hg19] 16p.11.2(29,580,020–30,177,999) × 1	BP4–5	de novo

SNP, single-nucleotide polymorphism; BP, breakpoint; na, not available.

**Table 2:** Clinical characteristics in Korean patients with 16p11.2 microdeletion syndrome.

Cases	S/A(y)	Neonatal Hypotonia	BIE *	DD/ID	ASD	Obesity	Vertebral Abnormalities
A-I-1	M/64	no	present	mild	no	present	no
A-II-1	F/36	no	no	mild	no	present	no
A-III-1	F/12	present	present	mild	no	present	scoliosis
A-III-4	M/6	present	no	moderate	no	present	scoliosis
B-II-2	M/5	present	present	moderate	present	no	scoliosis
C-II-2	M/16	present	present	mild	no	no	no
D-I-2	F/35	no	no	mild	no	present	no
D-II-1	M/3	present	no	moderate	no	present	vertebral dysplasia no
E-II-1	M/1	present	present	mild	no	no	vertebral dysplasia no
F-II-1	F/1	no	present	mild	no	no	no

S/A(y), sex/age(year); BIE, benign infantile epilepsy; DD/ID, developmental delay/intellectual disability; ASD, autism spectrum disorder. * BIE was effectively managed with anti-seizure medications, including oxcarbazepine and/or levetiracetam.

## Data Availability

Data are contained within the article.
